# 2,4-Dibromo-6-[(hydroxyimino)methyl]phenol

**DOI:** 10.1107/S1600536811028741

**Published:** 2011-07-23

**Authors:** Yang-Hui Luo, Jian Xu, Mei-Ling Pan, Jin-Feng Li

**Affiliations:** a Ordered Matter Science Research Center, College of Chemistry and Chemical, Engineering, Southeast University, Nanjing 210096, People’s Republic of China

## Abstract

In the title compound, C_7_H_5_Br_4_NO_2_, intra­molecular O—H⋯N hydrogen bonds are observed. In the crystal structure, inter­molecular O—H⋯O hydrogen bonds link the mol­ecules into dimers.

## Related literature

For details of the preparation, see: Dey *et al.* (2003 ▶). 
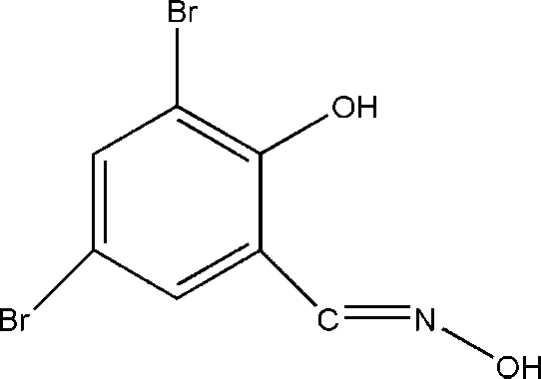


## Experimental

### 

#### Crystal data

C_7_H_5_Br_2_NO_2_
*M* *_r_* = 294.94
Triclinic, 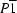

*a* = 4.2590 (5) Å
*b* = 8.6742 (7) Å
*c* = 12.0831 (11) Å
α = 74.171 (1)°
β = 82.248 (2)°
γ = 79.028 (1)°
*V* = 419.98 (7) Å^3^
*Z* = 2
Mo *K*α radiation
μ = 9.60 mm^−1^
*T* = 293 K
0.80 × 0.42 × 0.18 mm

#### Data collection

Rigaku R-AXIS RAPID CCD area-detector diffractometer
Absorption correction: multi-scan (*CrystalClear*; Rigaku, 2005 ▶) *T*_min_ = 0.048, *T*_max_ = 0.277
2162 measured reflections
1453 independent reflections
987 reflections with *I* > 2σ(*I*)
*R*_int_ = 0.037

#### Refinement

*R*[*F* ^2^ > 2σ(*F* ^2^)] = 0.063
*wR*(*F* ^2^) = 0.160
*S* = 1.05
1453 reflections
109 parameters
H-atom parameters constrained
Δρ_max_ = 1.50 e Å^−3^
Δρ_min_ = −1.30 e Å^−3^

### 

Data collection: *CrystalClear* (Rigaku, 2005 ▶); cell refinement: *CrystalClear*; data reduction: *CrystalClear*; program(s) used to solve structure: *SHELXS97* (Sheldrick, 2008 ▶); program(s) used to refine structure: *SHELXL97* (Sheldrick, 2008 ▶); molecular graphics: *ORTEPIII* (Burnett & Johnson, 1996 ▶), *ORTEP-3 for Windows* (Farrugia, 1997 ▶) and *PLATON* (Spek, 2009 ▶); software used to prepare material for publication: *SHELXL97*.

## Supplementary Material

Crystal structure: contains datablock(s) I, global. DOI: 10.1107/S1600536811028741/jh2311sup1.cif

Structure factors: contains datablock(s) I. DOI: 10.1107/S1600536811028741/jh2311Isup2.hkl

Supplementary material file. DOI: 10.1107/S1600536811028741/jh2311Isup3.cml

Additional supplementary materials:  crystallographic information; 3D view; checkCIF report

## Figures and Tables

**Table 1:** Hydrogen-bond geometry (Å, °)

*D*—H⋯*A*	*D*—H	H⋯*A*	*D*⋯*A*	*D*—H⋯*A*
O1—H1⋯O2^i^	0.82	2.10	2.775 (8)	140
O2—H2⋯N1	0.82	1.88	2.601 (10)	147

Symmetry code: (i) 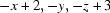.

**Fig. 1.**
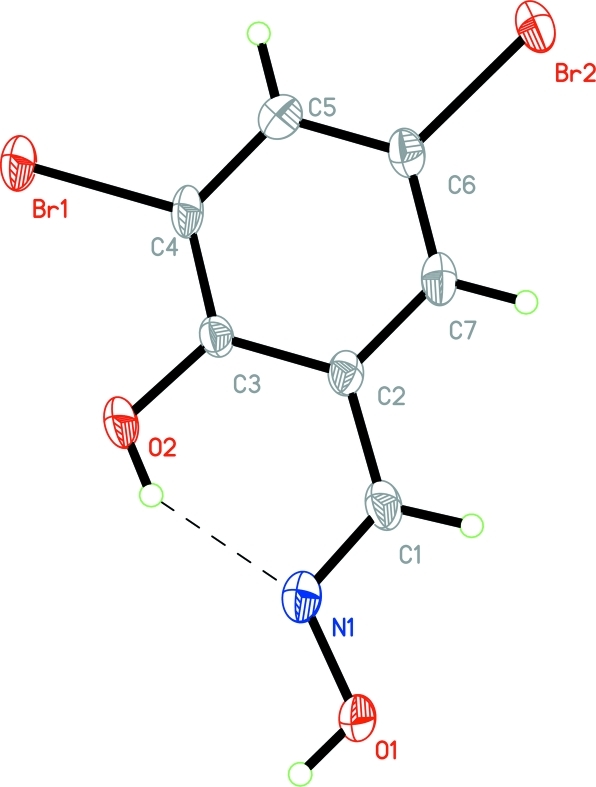
The molecular structure of the title compound with the atom-labelling scheme. Displacement ellipsoids are drawn at the 30% probability level. H atoms are represented as small spheres of arbitrary radii. Intramolecular hydrogen bonds are shown as dashed line.

**Fig. 2.**
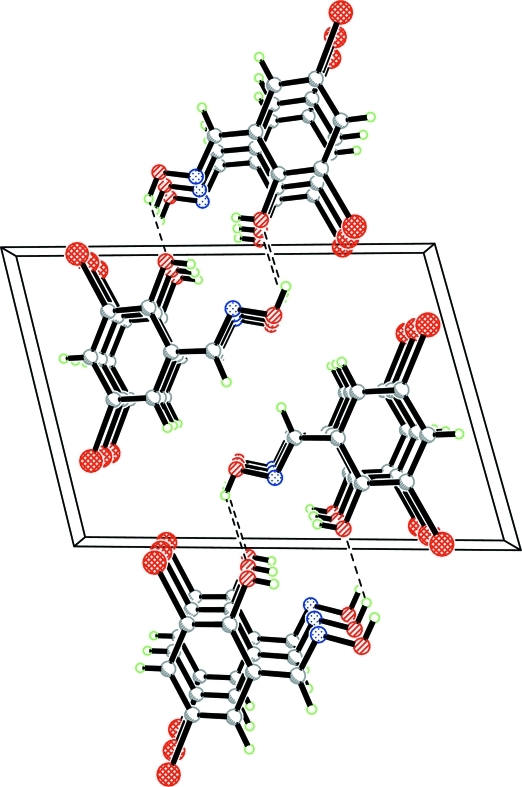
A packing view down the a axis showing the three dimensional network.Intermolecular hydrogen bonds are shown as dashed lines. Intramolecular O—H···N hydrogen bonds have been omitted for the sake of clarity.

## supplementary crystallographic information

### Comment

The derivatives of salicylaldehyde are important chemical materials, because they are excellent ligands for transition metals. As part of our interest in these ligands, we report here the crystal structure of the title compound.

The molecular structure of the title compound is shown in Fig. 1, where the dash line indicates the intramolecular O—H···N hydrogen bond.

All the non-H atoms of the title compound are located almost in one plane, as the atoms O1,O2 and N1 are shifted *ca* 0.1204 Å,0.0727Å and 0.0402Å out of the benzene ring plane, respectively.

The title compound formed dimer *via* intermolecular O—H···O hydrogen bonds and the dimers packed *via*π···π stacking interactions (3.4367 Å) (Fig. 2).

### Experimental

3,5-dibromosalicylaldoxime were synthesized as follows: 0.2 mol (13.9 g) hydroxylamine hydrochloride in companied with 0.2 mol (8 g) NaOH were dissolved in 50 ml ethanol solution in a 250 ml round bottomed flask and stirred to homogeneous. After that, an ethanol solution (30 ml) with 0.2 mol (40 g) 3,5-dibromosalicylicaldehyde was added dropwise to this solution at 70 °C and refluxed for about 2 h. After cooling and filtrating, crude compound of 3,5-dibromosalicylaldoximewas gained. Pure compound of it was obtained by crystallizing from 20 ml ethanol solution (Dey, *et al.*, 2003).

Crystals of 3,5-dibromosalicylaldoxime suitable for X-ray diffraction were obtained by slow evaporation of a methanol solution.

### Refinement

All H atoms attached to C atoms and O atoms were fixed geometrically and treated as riding with C—H = 0.93 Å (CH) and O—H = 0.82 Å with *U*_iso_(H) = 1.2*U*_eq_(C) and *U*_iso_(H) = 1.5*U*_eq_(O).

### Crystal data

**Table:**

C_7_H_5_Br_2_NO_2_	*Z* = 2
*M**_r_* = 294.94	*F*(000) = 280
Triclinic, *P*1	*D*_x_ = 2.332 Mg m^−^^3^
Hall symbol: -P 1	Mo *K*α radiation, λ = 0.71073 Å
*a* = 4.2590 (5) Å	Cell parameters from 808 reflections
*b* = 8.6742 (7) Å	θ = 2.5–26.6°
*c* = 12.0831 (11) Å	µ = 9.60 mm^−^^1^
α = 74.171 (1)°	*T* = 293 K
β = 82.248 (2)°	Prism, white
γ = 79.028 (1)°	0.80 × 0.42 × 0.18 mm
*V* = 419.98 (7) Å^3^	

### Data collection

**Table:**

Rigaku R-AXIS RAPID CCD area-detector diffractometer	1453 independent reflections
Radiation source: fine-focus sealed tube	987 reflections with *I* > 2σ(*I*)
graphite	*R*_int_ = 0.037
Detector resolution: 8.192 pixels mm^-1^	θ_max_ = 25.0°, θ_min_ = 2.5°
φ and ω scans	*h* = −5→5
Absorption correction: multi-scan (*CrystalClear*; Rigaku, 2005)	*k* = −9→10
*T*_min_ = 0.048, *T*_max_ = 0.277	*l* = −13→14
2162 measured reflections	

### Refinement

**Table:**

Refinement on *F*^2^	Primary atom site location: structure-invariant direct methods
Least-squares matrix: full	Secondary atom site location: difference Fourier map
*R*[*F*^2^ > 2σ(*F*^2^)] = 0.063	Hydrogen site location: inferred from neighbouring sites
*wR*(*F*^2^) = 0.160	H-atom parameters constrained
*S* = 1.05	*w* = 1/[σ^2^(*F*_o_^2^) + (0.0812*P*)^2^] where *P* = (*F*_o_^2^ + 2*F*_c_^2^)/3
1453 reflections	(Δ/σ)_max_ = 0.001
109 parameters	Δρ_max_ = 1.50 e Å^−^^3^
0 restraints	Δρ_min_ = −1.30 e Å^−^^3^

### Special details

**Table:**

Geometry. All e.s.d.'s (except the e.s.d. in the dihedral angle between two l.s. planes) are estimated using the full covariance matrix. The cell e.s.d.'s are taken into account individually in the estimation of e.s.d.'s in distances, angles and torsion angles; correlations between e.s.d.'s in cell parameters are only used when they are defined by crystal symmetry. An approximate (isotropic) treatment of cell e.s.d.'s is used for estimating e.s.d.'s involving l.s. planes.
Refinement. Refinement of *F*^2^ against ALL reflections. The weighted *R*-factor *wR* and goodness of fit *S* are based on *F*^2^, conventional *R*-factors *R* are based on *F*, with *F* set to zero for negative *F*^2^. The threshold expression of *F*^2^ > σ(*F*^2^) is used only for calculating *R*-factors(gt) *etc*. and is not relevant to the choice of reflections for refinement. *R*-factors based on *F*^2^ are statistically about twice as large as those based on *F*, and *R*- factors based on ALL data will be even larger.

### Fractional atomic coordinates and isotropic or equivalent isotropic displacement parameters (Å^2^)

**Table:**

	*x*	*y*	*z*	*U*_iso_*/*U*_eq_	
Br1	0.3846 (3)	0.03675 (11)	1.18327 (9)	0.0501 (4)	
Br2	0.1284 (3)	0.71718 (11)	1.09089 (9)	0.0508 (4)	
N1	0.9050 (19)	0.2301 (9)	1.5027 (7)	0.040 (2)	
O1	1.0649 (17)	0.2544 (8)	1.5855 (6)	0.0474 (18)	
H1	1.1377	0.1664	1.6269	0.071*	
O2	0.6873 (17)	0.0731 (7)	1.3820 (6)	0.0469 (18)	
H2	0.7721	0.0846	1.4357	0.070*	
C1	0.783 (2)	0.3610 (10)	1.4352 (8)	0.037 (2)	
H1A	0.8077	0.4594	1.4471	0.045*	
C2	0.609 (2)	0.3625 (10)	1.3421 (8)	0.031 (2)	
C3	0.575 (2)	0.2192 (10)	1.3157 (7)	0.030 (2)	
C4	0.424 (2)	0.2277 (10)	1.2216 (8)	0.036 (2)	
C5	0.287 (2)	0.3756 (11)	1.1518 (8)	0.040 (2)	
H5	0.1827	0.3798	1.0881	0.048*	
C6	0.314 (2)	0.5157 (10)	1.1813 (8)	0.036 (2)	
C7	0.477 (2)	0.5089 (11)	1.2733 (8)	0.038 (2)	
H7	0.4994	0.6049	1.2896	0.045*	

### Atomic displacement parameters (Å^2^)

**Table:**

	*U*^11^	*U*^22^	*U*^33^	*U*^12^	*U*^13^	*U*^23^
Br1	0.0685 (8)	0.0273 (6)	0.0611 (8)	−0.0064 (5)	−0.0103 (6)	−0.0208 (5)
Br2	0.0661 (8)	0.0263 (6)	0.0574 (8)	0.0040 (5)	−0.0175 (5)	−0.0084 (5)
N1	0.044 (5)	0.031 (4)	0.048 (5)	−0.005 (4)	−0.002 (4)	−0.016 (4)
O1	0.063 (5)	0.026 (3)	0.058 (5)	0.004 (3)	−0.025 (4)	−0.017 (3)
O2	0.067 (5)	0.019 (3)	0.055 (4)	0.003 (3)	−0.015 (4)	−0.012 (3)
C1	0.031 (5)	0.024 (5)	0.055 (6)	0.003 (4)	−0.005 (4)	−0.012 (5)
C2	0.022 (5)	0.026 (5)	0.048 (6)	0.002 (4)	−0.002 (4)	−0.017 (4)
C3	0.038 (5)	0.021 (4)	0.030 (5)	−0.001 (4)	−0.003 (4)	−0.008 (4)
C4	0.037 (5)	0.022 (5)	0.051 (6)	−0.003 (4)	0.004 (5)	−0.019 (4)
C5	0.043 (6)	0.040 (6)	0.040 (6)	−0.008 (5)	−0.006 (5)	−0.014 (5)
C6	0.044 (6)	0.026 (5)	0.038 (5)	−0.001 (4)	0.001 (4)	−0.011 (4)
C7	0.036 (6)	0.025 (5)	0.053 (6)	−0.008 (4)	0.003 (5)	−0.013 (4)

### Geometric parameters (Å, °)

**Table:**

Br1—C4	1.879 (8)	C2—C7	1.377 (13)
Br2—C6	1.882 (9)	C2—C3	1.402 (11)
N1—C1	1.268 (12)	C3—C4	1.360 (13)
N1—O1	1.364 (10)	C4—C5	1.397 (13)
O1—H1	0.8200	C5—C6	1.384 (12)
O2—C3	1.339 (10)	C5—H5	0.9300
O2—H2	0.8200	C6—C7	1.371 (13)
C1—C2	1.424 (13)	C7—H7	0.9300
C1—H1A	0.9300		
			
C1—N1—O1	113.3 (7)	C3—C4—C5	122.1 (8)
N1—O1—H1	109.5	C3—C4—Br1	120.1 (7)
C3—O2—H2	109.5	C5—C4—Br1	117.8 (7)
N1—C1—C2	122.3 (8)	C6—C5—C4	117.4 (9)
N1—C1—H1A	118.9	C6—C5—H5	121.3
C2—C1—H1A	118.9	C4—C5—H5	121.3
C7—C2—C3	118.5 (9)	C7—C6—C5	121.0 (9)
C7—C2—C1	119.4 (8)	C7—C6—Br2	120.3 (7)
C3—C2—C1	122.0 (8)	C5—C6—Br2	118.7 (8)
O2—C3—C4	119.1 (8)	C6—C7—C2	121.2 (8)
O2—C3—C2	121.3 (8)	C6—C7—H7	119.4
C4—C3—C2	119.7 (8)	C2—C7—H7	119.4
			
O1—N1—C1—C2	179.2 (7)	C2—C3—C4—Br1	178.5 (6)
N1—C1—C2—C7	178.8 (9)	C3—C4—C5—C6	0.6 (14)
N1—C1—C2—C3	−3.0 (14)	Br1—C4—C5—C6	179.4 (6)
C7—C2—C3—O2	−177.4 (8)	C4—C5—C6—C7	2.0 (14)
C1—C2—C3—O2	4.4 (13)	C4—C5—C6—Br2	−179.0 (7)
C7—C2—C3—C4	2.2 (13)	C5—C6—C7—C2	−2.5 (14)
C1—C2—C3—C4	−176.0 (8)	Br2—C6—C7—C2	178.6 (7)
O2—C3—C4—C5	176.9 (8)	C3—C2—C7—C6	0.3 (13)
C2—C3—C4—C5	−2.7 (14)	C1—C2—C7—C6	178.6 (8)
O2—C3—C4—Br1	−1.9 (12)		

### Hydrogen-bond geometry (Å, °)

**Table:**

*D*—H···*A*	*D*—H	H···*A*	*D*···*A*	*D*—H···*A*
O1—H1···O2^i^	0.82	2.10	2.775 (8)	140.
O2—H2···N1	0.82	1.88	2.601 (10)	147.

Symmetry codes: (i) −*x*+2, −*y*, −*z*+3.
